# CMRI morphological and functional changes of the heart in myocarditis and dilated cardiomyopathy in acute pediatric heart failure

**DOI:** 10.1186/1532-429X-18-S1-P251

**Published:** 2016-01-27

**Authors:** Ahmed E Kharabish

**Affiliations:** grid.7776.10000000406399286Radiology, Cairo Universty, Cairo, Egypt

## Background

Our purpose is to find morphological and functional changes of the heart in acute heart failure presentation using cardiac magnetic resonance (CMRI) in pediatric group in order to differentiate between dilated cardiomyopathy (DCM) and myocarditis as a cause of acute heart failure.

## Methods

Twenty patients presented with acute heart failure. Echo and cardiac markers (including Troponin, CK, CK-MB) were performed for every patient. Patient's age ranged from 2 mo-14years. All patients underwent CMRI study. All CMRI studies were scanned using the routine protocol to fulfill the Lake Louise criteria. The performed sequences include: 1.cine stead state free precession in axial and short axis to calculate cardiac function and volumes, 2.T2 TSE in short axis cuts for cardiac edema, the cut off value of >2.3 obtained from the ratio between myocardial muscle to skeletal muscle signals was used, 3. Short axis T1 TSE before and 5 minutes after contrast injection was used as marker of capillary leakage, and delayed myocardial enhancement (LGE) in short axis and four chamber images to show the LGE pattern. Children under 6 years were sedated with chloral hydrate.

## Results

The echo and CMRI showed in all patients hypokinetic left ventricle (LV) with dilated indexed end diastolic volume (EDVI). Echo reports were positive to myocarditis in 4 patients. Cardiac markers were high in 8 patients. In patients with negative CMRI-Lake Louise criteria (12 patients) and negative cardiac markers; the average LVEDVI amounted to 159ml/m² and the average of LV ejection fraction amounted to 22%. In patients with positive CMRI lake Louise criteria (8 patients) the average LVEDVI amounted to 132ml/m² and the average of LV ejection fraction amounted to 22%. LGE in the positive cases of myocarditis was extensive and diffuse. The lateral wall was involved in all positive myocarditis patients. No LGE, edema or evidence of capillary leakage was found in DCM patients.

## Conclusions

LV end diastolic volumes were higher in DCM patients who had negative Lake Louise criteria and negative labs markers than in positive myocarditis patients. EF% may be similar during the acute phase in both types of patients whether positive or negative to myocarditis. A prospective CMRI study will be designed to follow the changes of the EF and DE-CMRI of the same patients.

CMRI is very important tool in differentiating the causes of acute heart failure in pediatric group especially in our developing country. The CMRI Lake Louise criterion together with lab findings increased accuracy of diagnosing those patients, which saved treatment cost and biopsies.

**Figure 1:**
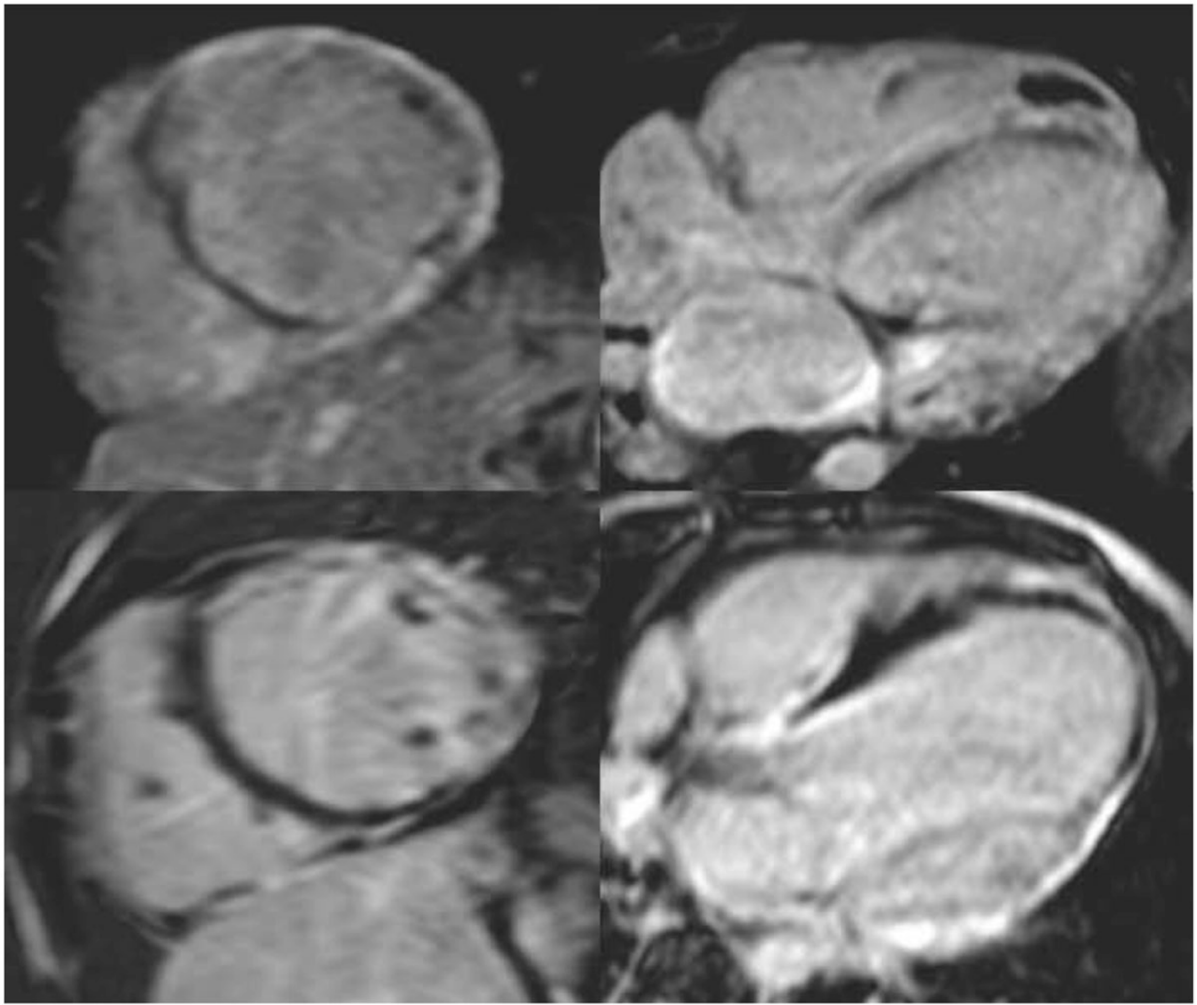
**Cardiac MRI late Gadolinium enhancement, short axis view (right column) and four chamber views (left column) showing diffuse epi-cardial and transmural involvement in two different cases of myocarditis**. Upper row are images of same case and lower row are of the second case.

